# Does unintended birth lead to zero dose of DPT vaccine among children aged 12–23 months in India?

**DOI:** 10.1080/21645515.2024.2417526

**Published:** 2024-11-07

**Authors:** Pritu Dhalaria, Pawan Kumar, Ajay Verma, Pretty Priyadarshini, Ajeet Kumar Singh, Bhupendra Tripathi, Arindam Ray

**Affiliations:** a Immunization Technical Support Unit, Ministry of Health & Family Welfare, Government of India, New Delhi, India; b Immunization Division, Ministry of Health & Family Welfare, New Delhi, India; c Department of Economics, Banaras Hindu University, Varanasi, Uttar Pradesh, India; d Bill & Melinda Gates Foundation, New Delhi, India

**Keywords:** Unintended birth, DPT, zero-dose, association, vaccination, India, immunization agenda 2030

## Abstract

Zero-dose children pose a key challenge in immunization programs due to their association with access to the health system and primary healthcare services. Examining zero-dose aids an in-depth understanding of healthcare disparities among children and caregivers. The disparity in utilization of maternal and child health services raises concerns about the potential consequences of unintended pregnancies on vaccine uptake. The National Family Health Survey 2019–21 (NFHS-5) served as the data source, and the study analyzed information from 43,247 children aged 12–23 months. Sociodemographic variables such as birth order, wealth quintile, gender, social group, religion, residence, mother education, and delivery-related factors were considered. Statistical analysis involved weighted estimates, chi-square tests, and multivariate multinomial logistic regression. The results show that 9.14% of children from unintended pregnancies were zero-dose for the DPT vaccine, compared to only 6.69% of children from intended pregnancies in India, indicating a higher prevalence of zero-dose associated with unintended pregnancies. The regression analysis shows the adjusted odds among children from an unintended birth − 1.21 times higher for the zero-dose DPT vaccine as compared to the intended birth (AOR: 1.21, 95% CI: 1.06,1.38). Zero-dose immunization has become a crucial metric of childhood immunization performance, gaining prominence in national agendas, the IA 2030 framework, and Gavi’s 2021–25 strategy. The study findings highlight a significant association between unintended pregnancy and zero-dose DPT vaccination. The results provide compelling evidence that unintended pregnancies could be a potential risk factor for zero-dose DPT vaccination in low- and middle-income countries.

## Introduction

Immunization programs have been established as one of the most cost-effective public health interventions for childhood disease prevention and health promotion.^1^ The immunization program protects a person against vaccine-preventable infectious diseases, reducing the risk of a particular disease across life course.^2^ Vaccination of children under 24  months is critical in preventing life-threatening diseases and contributes to longer and healthier lives for individuals of all ages.^2–4^ Vaccines have significantly impacted the incidence of VPDs worldwide and decreased childhood morbidity and mortality rates.

The under-5 mortality in children has significantly decreased over the years. From 9.7 million in 2000, it has reduced to 4.9 million in 2022 – a 51% reduction.^5^ This can be attributed to the prevention and control of various diseases, with four of the top 10 causes being either fully or partially preventable through vaccination – diarrhea, pneumonia, meningitis, and measles.^6,7^ Annually, immunization programs avert 3.5–5.0 million fatalities caused by various vaccine-preventable illnesses.^8^ Despite this global progress, approximately 14.3 million children worldwide missed out on receiving their first Diphtheria Tetanus Pertussis (DPT) vaccine dose, and 6.2 million were not fully vaccinated in 2022. Most of these children reside in low- and middle-income countries (LMICs).^9^

Global Alliance for Vaccines and Immunization’s (GAVI) 5.0 strategy (2021–25) targets zero-dose children and missed communities to achieve the child health indicators outlined in the Sustainable Development Goals (SDGs) 2030 and aligned with the Immunization Agenda 2030 (IA 2030).^10^ Zero-dose are those children who have not received any doses of diphtheria, tetanus, and pertussis-containing vaccines by 12 months of age.^11^ This definition of zero-dose is for operational purposes and has been used throughout this paper. About two-thirds (~65%) of zero-dose children live in merely 10 countries – Angola, Brazil, Democratic Republic of the Congo, Ethiopia, India, Indonesia, Mexico, Nigeria, Pakistan, and the Philippines.^12^ India contributes 17.6% of the world’s total cohort of surviving infants.^13^ The higher number of zero-dose children in India is also attributed to a relatively higher birth cohort and even a small percentage of children translates into huge absolute numbers as compared to other LMICs. In 2022, India’s contribution to the global zero-dose burden was reduced significantly to 7.9% from 14.9% in 2021.^14^ The presence of zero-dose children remains a concern for countries and policymakers. These children represent an inadequate population immunity against VPDs and underscore the disparities in coverage of essential health services worldwide, especially in LMICs.^15,16^

The primary objective of IA 2030 is to ensure equitable access to essential vaccines and eliminate gaps in immunization coverage. The IA 2030 and the Gavi 5.0 have established global targets to reduce the number of zero-dose children. These targets aim to decrease the number of children who have not received any vaccinations by 25% by 2025 and by 50% by 2030, aligning with the SDGs 2030.^17^ To reach zero-dose and under-vaccinated children, IA 2030 envisages a global strategy to ‘leave no one behind’ and to achieve the global vision of a world where everyone, everywhere, at every age fully benefits from vaccines for good health and well-being. The IA 2030 thus stands as a vision for our current and forthcoming world wherein vaccines are readily available and accessible, offering to safeguard against diseases, promoting wellness, and enabling life opportunities for all, regardless of the circumstances of their birth uncertainties, geography, or economic status.^18^ This vision is collaboratively crafted through contributions from diverse segments of the global community, stakeholders, and beneficiaries.^7^ IA 2030, with its seven strategic priorities, emphasizes understanding the social factors that influence vaccine uptake and confidence to enhance the coverage of basic vaccination. This includes addressing social processes, gender-related barriers, practical considerations, and combating misinformation or disinformation.^19^

India introduced the Expanded Programme on Immunization (EPI) in 1978, which was later transformed into the Universal Immunization Program (UIP) in 1985 to reduce morbidity and mortality caused by major VPDs.^20^ The immunization program was implemented through a network of healthcare facilities – primary health centers, sub-centers, community health centers, Anganwadi centers/day-care, and hospitals across the country. So far, India has achieved 76.4% Full Immunization Coverage (FIC) for all basic vaccinations among children of 12–23 months and 20.0% of children partially vaccinated. In addition, the National Family Health Survey (NFHS) 5 report from 2019 to 2021 recorded that 6.8% of children aged 12–23 months did not receive the first dose of the DPT vaccine, accounting for more than 2.7 million children in India.^21,22^

Previous studies have addressed the prevalence trend, pattern, barriers, and challenges of zero-dose children for the DPT vaccine across different countries.^17,23,24^ Past studies in India describe the pattern and prevalence of zero-doses of DPT vaccine across the Indian states between 1993 and 2021.^25^ Key sociodemographic variables and equity determinants – lowest wealth quintile, children of mothers with low levels of education, fewer Ante Natal Care (ANC) visits, and no institutional birth were significantly associated with higher odds for zero-dose of DPT vaccine among children 12–23 months.^23,24,26–28^ In addition, some other studies from LMICs have also considered socioeconomic and gender-related inequalities in zero-dose children. Maternal and child health services such as antenatal care, institutional deliveries, and modest healthcare-seeking behavior were associated with inequalities in zero doses of the DPT vaccine.^29^ One study analyzed data from 80 LMICs in which the zero-dose was strongly associated with childhood stunting, lower access to improved water and sanitation, lower levels of maternal education, and lower levels of demand-satisfied for family planning with modern methods.^30^

Various studies highlight that children born from unintended pregnancies suffer from different social and health problems.^31,32^ These studies consistently reflect the critical issue of utilization of maternal and child health services in the context of pregnancy intendedness. Unintended pregnancy is linked to adverse infant outcomes like preterm birth and low birth weight, potentially affecting child health and utilization of healthcare services. Unintended pregnancies are linked to poor child health outcomes – including deliveries without skilled attendants, incomplete immunization, and child stuntedness, leading to higher child mortality rates.^33–37^ The mothers of these children did not receive adequate prenatal care and subsequently were devoid of information regarding immunization for their children. Despite the wealth of studies, there remain notable limitations in our understanding of this relationship. Moreover, primary evidence underscores the significant impact of unintended pregnancies on maternal and child health outcomes. While many factors affect the rate of vaccination in children, unintended pregnancy is an important indicator that influences vaccination rates and there is a need to explore this relationship. Most of the existing research and literature focuse on the prevalence and determinants of zero-dose of the DPT; however, our study goes one step further and examines the association of zero-dose prevalence for children from the lens of ‘pregnancy intendedness.’

Zero-dose children pose a key challenge in immunization programs due to their association with access to the health system and various primary healthcare services, aiding in understanding healthcare disparities among children and caregivers. The disparity in utilization of maternal and child health services raises concerns about the potential consequences of unintended pregnancies on low vaccine uptake. This study attempts to bridge the knowledge gap by exploring the association between community, individual, household level, and social factors that could be associated with zero-dose of the DPT vaccine using NFHS-5 data. This analysis examines the association between births resulting from pregnancy intendedness and zero-dose of the DPT vaccine among children aged 12–23 months in India.

## Materials and methodology

The present study uses data from the National Family Health Survey 2019–21 (NFHS-5) – a nationally representative household-level cross-sectional survey for India. NFHS-5 provides information on population health, nutrition, and child health indicators by background characteristics at the national, state, and district levels to assist policymakers and program managers in examining progress over time and ensuring it supports data-driven decision-making for the Ministry of Health and Family Welfare (MoHFW) and Government of India, which is available in the public domain and permission is not required to re-analyze and publish the data. All five rounds of the NFHS have been conducted under the leadership of the Ministry of Health and Family Welfare, Government of India. The primary objective of each round has been to provide high-quality data on health, family welfare, and emerging issues in this domain. This survey adopted a stratified two-stage sample design. It was executed in two distinct phases – the first phase spanned from June 17, 2019, to January 30, 2020, encompassing 17 states and 5 Union Territories (UTs), while the second phase began on November 2020 to April 2021, covering 11 states and 3 UTs. In total, NFHS-5 collected data from 636,699 households, 724,115 women, and 101,839 men. For this study, a sample of 43,247 children aged 12–23 months were considered.

### Outcome and exposure variables

The NFHS-5 seeks information regarding all basic vaccinations (BCG, OPV, Penta, measles) provided under the UIP for children aged 12–23 months at the time of the survey. Children who have not received any of these vaccines are classified as zero-dose. The outcome of interest for this study is the prevalence of the zero-dose DPT vaccine among children aged 12–23 months in India between 2019 and 2021. This analysis aims to investigate the factors of interest influencing zero-dose occurrence in children. The characteristics of the variables are outlined in Table 1.

**Table 1.** Characteristics of the variable for analysis.

Received DPT	Percentage	[95%, C.I]	Sample
No	6.88	[6.55,7.22]	2974
Yes	93.12	[92.78,93.45]	40273
**Unintended birth**			
No	92.26	[91.90,92.61]	39900
Yes	7.74	[7.39,8.10]	3347
**Gender**			
Male	52	[51.35,52.65]	22489
Female	48	[47.35,48.65]	20758
**Wealth Index**			
Richest	16.13	[15.62,16.65]	6974
Richer	18.75	[18.22,19.30]	8110
Middle	19.86	[19.34,20.39]	8590
Poorer	21.36	[20.86,21.88]	9238
Poorest	23.9	[23.38,24.42]	10335
**Birth Order**			
1	39.71	[39.07,40.35]	17171
2-3	49.21	[48.56,49.86]	21283
4 and more	11.08	[10.70,11.47]	4793
**Mother education in years**			
12 or more years	17.24	[16.72,17.78]	7456
9-12 year	34.7	[34.08,35.32]	15006
5-8 years	24.66	[24.11,25.22]	10663
<5 years	4.36	[4.11,4.63]	1886
No schooling	19.04	[18.57,19.53]	8236
**Social group**			
None of them	23.58	[22.99,24.18]	10198
OBC	43.3	[42.66,43.94]	18724
Schedule tribe	10	[9.64,10.37]	4326
Schedule caste	23.12	[22.58,23.67]	9999
**Religion**			
Hindu	79.56	[79.01,80.10]	34407
Muslim	16.13	[15.63,16.64]	6974
Christian	1.98	[1.82,2.16]	857
Others	2.33	[2.16,2.52]	1008
**Type Of Place of Residence**			
Urban	26.9	[26.25,27.55]	11632
Rural	73.1	[72.45,73.75]	31614
**Place of delivery**			
Private health facility	63.06	[62.42,63.70]	27272
Public health facility	27.51	[26.89,28.13]	11896
No health facility	9.43	[9.08,9.79]	4079
**Sex Of Household Head**			
Male	85.04	[84.57,85.48]	36775
Female	14.96	[14.52,15.43]	6472
**Residing with husband**			
Living with him	86.52	[86.10,86.94]	37418
Staying elsewhere	13.48	[13.06,13.90]	5829
**Total**			**43247**

The exposure variable of interest selected for this analysis – is whether the childbirth is from an intended or unintended pregnancy. The NFHS collects data on fertility preference from women whose children were born in the last 5 years – whether the pregnancy was wanted (planned birth) at a later time (mistimed birth) or not at all planned (unwanted birth) at the time of conception. The variables were categorized into two – intended (wanted and planned) and unintended pregnancy (mistimed birth and unwanted birth). The analysis included only live intended and unintended births, which were currently aged 12–23 months old. Other socio-demographic factors that affect routine vaccination coverage are – gender, wealth index, birth order, level of mother’s education in years, social groups, religion, place of residence, place of delivery, sex of household head, and whether the woman is residing with her husband.^2,33,36,37^

This study employs bivariate analysis using the chi-square test to determine the zero-dose DPT vaccine uptake percentage with pregnancy intention and control variables. Additionally, bivariate logistic regression is used to examine the unadjusted association between pregnancy intention and zero-dose DPT vaccination. Further, this study uses multivariate logistic regression to determine the adjusted association between pregnancy intendedness and zero-dose. The Propensity Score Matching (PSM) method was employed to conduct causal analyses to compare zero-dose children for the DPT vaccine among children born from intended pregnancy and unintended pregnancy. This analysis uses the k-match command to estimate the difference with kernel matching function in zero-dose children for the DPT vaccine between the untreated and control, and it is estimated by average treatment effect (ATE) and average treatment effect on the treated (ATT).^38^ The balancing properties of the kernel matching were satisfied. It substantially reduces covariate bias, as illustrated in Figure 2. The data were analyzed using statistical software – Stata version 16.0 SE.

## Results

**Figure 1.**
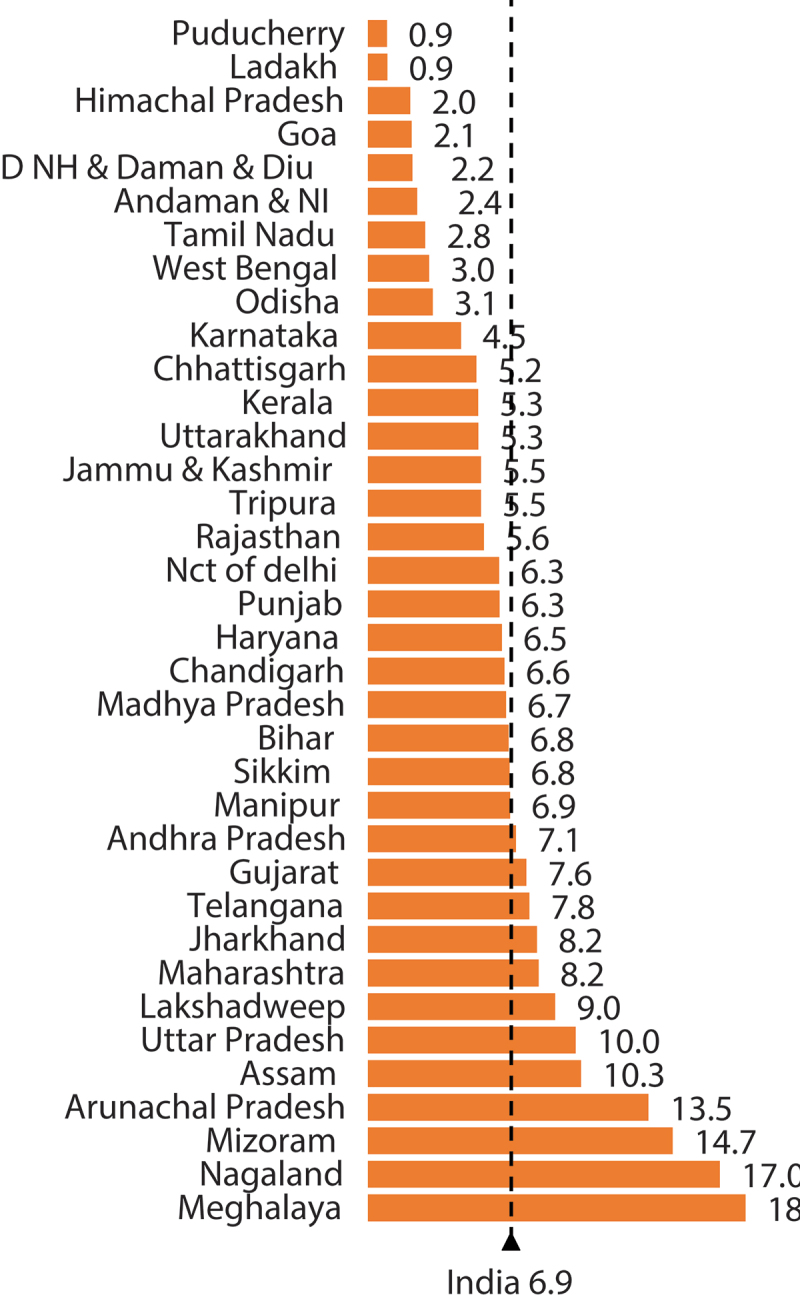
Percentage of children aged 12–23 who did not receive DPT by state/union territory, India, 2019–21.

The result of bivariate analysis (Table 2) shows that among children of unintended pregnancy, 9.14% were zero-dose for DPT as compared to only 6.69% zero-dose children of intended pregnancy in India – higher prevalence associated with unintended pregnancy. Figure 1 shows that the percentage of zero-dose for DPT ranges between 0.9% and 18% across 36 states and UTs of India. While only 1% of children aged 12–23 months did not receive the first dose of the DPT vaccine in Puducherry, a huge proportion of 18% of children aged 12–23 months did not receive it in Meghalaya state in India depicting huge inequities.

**Table 2.** Percentage of children aged 12–23 who have not received DPT vaccine by background characteristics, India, 2019–21.

Background variable	Zero-dose of DPT vaccine		Chi2 (p < .000)	Sample
	YES	NO		
**Unintended birth**	**% [95%, C.I]**	**% [95%, C.I]**		
No	6.69, [6.36,7.03]	93.31, [92.97,93.64]	29.21(0.003)	39900
Yes	9.14, [7.47,11.15]	90.86, [88.85,92.53]		3347
**Gender**				
Male	6.48, [6.06,6.92]	93.52, [93.08,93.94]	11.85(0.015)	22489
Female	7.31, [6.80,7.86]	92.69, [92.14,93.20]		20758
**Wealth Index**				
Richest	5.94, [5.13,6.87]	94.06, [93.13,94.87]	130.82(0.000)	6974
Richer	5.47, [4.80,6.23]	94.53, [93.77,95.20]		8110
Middle	5.8, [5.08,6.63]	94.2, [93.37,94.92]		8590
Poorer	7.38, [6.65,8.17]	92.62, [91.83,93.35]		9238
Poorest	9.06, [8.39,9.77]	90.94, [90.23,91.61]		10335
**Birth Order**				
1 ^Ref.^	5.94, [5.48,6.44]	94.06, [93.56,94.52]	106.04(0.000)	17171
2-3	6.88, [6.38,7.42]	93.12, [92.58,93.62]		21283
4 and more	10.19, [9.19,11.29]	89.81, [88.71,90.81]		4793
**Mother education in years**				
12 or more years	5.18, [4.51,5.94]	94.82, [94.06,95.49]	249.35(0.000)	7456
9-12 year	5.71, [5.20,6.26]	94.29, [93.74,94.80]		15006
5-8 years	6.79, [6.05,7.61]	93.21, [92.39,93.95]		10663
<5 years	6.92, [5.68,8.40]	93.08, [91.60,94.32]		1886
No schooling	10.64, [9.84,11.51]	89.36, [88.49,90.16]		8236
**Social group**				
None of them	7.59, [6.81,8.46]	92.41, [91.54,93.19]	20.14(0.018)	10198
OBC	6.46, [6.01,6.93]	93.54, [93.07,93.99]		18724
Schedule tribe	7.75, [6.91,8.67]	92.25, [91.33,93.09]		4326
Schedule caste	6.56, [5.85,7.34]	93.44, [92.66,94.15]		9999
**Religion**				
Hindu	6.26, [5.92,6.63]	93.74, [93.37,94.08]	123.31(0.000)	34407
Muslim	9.9, [8.87,11.03]	90.1, [88.97,91.13]		6974
Christian	8.02, [6.48,9.90]	91.98, [90.10,93.52]		857
Others	5.98, [4.05,8.73]	94.02, [91.27,95.95]		1008
**Place Of Residence**				
Urban	7.85, [7.01,8.79]	92.15, [91.21,92.99]	23.86(0.003)	11632
Rural	6.52, [6.20,6.85]	93.48, [93.15,93.80]		31614
**Place of delivery**				
Private health facility	5.91, [5.54,6.31]	94.09, [93.69,94.46]	310.6(0.000)	27272
Public health facility	6.85, [6.16,7.62]	93.15, [92.38,93.84]		11896
No health facility	13.39, [12.13,14.75]	86.61, [85.25,87.87]		4079
**Sex of Household Head**				
Male	6.87, [6.51,7.25]	93.13, [92.75,93.49]	0.01(0.941)	36775
Female	6.91, [6.14,7.76]	93.09, [92.24,93.86]		6472
**Residing with husband**				
Living with him	6.71, [6.35,7.08]	93.29, [92.92,93.65]	12.11(0.009)	37418
Staying elsewhere	7.95, [7.08,8.91]	92.05, [91.09,92.92]		5829
**Total**	**6.88, [6.55,7.22]**	**93.12, [92.78,93.45]**		**43247**

Table 3 shows the result of the univariate and multivariate logistic regression of zero-dose DPT vaccine by potential factors among children aged 12–23 months in India. In model 1, univariate logistic regression reveals that the odds were higher for zero-dose DPT vaccine (OR: 1.33, 95% CI: 1.17,1.51) than for children who had an unintended birth compared to those from wanted or intended birth. Similarly, in model 2, among children from an unintended birth, the adjusted odds were 1.21 times higher for the zero-dose DPT vaccine compared to the wanted birth (AOR: 1.21, 95% CI: 1.06,1.38). Children from the poorest households were 1.69 times more likely to be zero-dose for the DPT vaccine than children from the richest households (AOR: 1.69, 95% CI: 1.42,2.00). Children whose mothers had not done any schooling were 1.50 times more likely to be zero-dose than children whose mothers had 12 or more years of education (AOR: 1.50, 95% CI: 1.28,1.76). Children who belonged to the ‘Other Backward Class’ (OBC) social group were 0.88 times less likely to be zero-dose for the DPT vaccine as compared with children of ‘General’ social groups (AOR: 0.88, 95% CI: 0.79,0.97). Children from the Christian and Muslim communities were 1.87 and 1.48 times, respectively, more likely to be zero-dose as compared to children from Hindu communities (AOR: 1.87, 95% CI: 1.62,2.14). Children residing in rural areas were 0.70 times less likely to be zero-dose than those living in urban areas (AOR: 0.70, 95% CI: 0.63,0.78). For institution-based deliveries, those children who were not delivered at any health facility were 2.16 times more likely to be zero-dose than children delivered at private health facilities (AOR: 2.16, 95% CI: 1.95,2.38). Children from those households with a female as a head were 0.83 times less likely to be zero-dose compared to children from those households with a male head (AOR: 0.83, 95% CI: 0.74,0.93). Children whose fathers were not living with them were 1.22 times more likely to be zero-dose than children whose fathers were living with them (AOR: 1.22, 95% CI: 1.09,1.37).

**Table 3.** Univariate (model 1) and multivariate logistic regression (model 2) on zero-dose of DPT by background variable.

Background variable	Model 1		Model 2	
Unintended Birth	OR	[95%, C.I]	AOR	[95%, C.I]
No^Ref.^	1		1	
Yes	1.33***	[1.17,1.51]	1.21**	[1.06,1.38]
**Gender**				
Male^Ref.^	1		1	
Female	1.08*	[1.01,1.16]	1.06	[0.99,1.14]
**Wealth Index**				
Richest^Ref.^	1		1	
Richer	1	[0.86,1.16]	1.07	[0.91,1.25]
Middle	1.12	[0.97,1.30]	1.22*	[1.04,1.44]
Poorer	1.48***	[1.30,1.69]	1.50***	[1.27,1.76]
Poorest	1.99***	[1.75,2.26]	1.69***	[1.42,2.00]
**Birth Order**				
1^Ref.^	1		1	
2-3	1.12**	[1.03,1.22]	0.98	[0.90,1.07]
4 and more	1.94***	[1.74,2.15]	1.11	[0.99,1.25]
**Mother education in years**				
12 or more years^Ref.^	1		1	
9-12 year	1.1	[0.97,1.25]	1	[0.87,1.15]
5-8 years	1.43***	[1.26,1.63]	1.14	[0.98,1.32]
<5 years	1.99***	[1.67,2.38]	1.24*	[1.01,1.51]
No schooling	2.20***	[1.94,2.51]	1.50***	[1.28,1.76]
**Social group**				
General^Ref.^	1		1	
OBC	0.84***	[0.76,0.93]	0.88*	[0.79,0.97]
Schedule tribe	1.40***	[1.26,1.55]	0.99	[0.86,1.13]
Schedule caste	0.86*	[0.77,0.97]	0.9	[0.79,1.02]
**Religion**				
Hindu^Ref.^	1		1	
Muslim	1.69***	[1.54,1.86]	1.48***	[1.33,1.65]
Christian	2.49***	[2.23,2.77]	1.87***	[1.62,2.14]
Others	1.19	[0.99,1.43]	1.19	[0.98,1.45]
**Place of Residence**				
Urban^Ref.^	1		1	
Rural	0.96	[0.88,1.05]	0.70***	[0.63,0.78]
**Place of delivery**	1			
Private health facility^Ref.^			1	
Public health facility	1.08	[0.98,1.19]	1.30***	[1.18,1.44]
No health facility	2.97***	[2.71,3.25]	2.16***	[1.95,2.38]
**Sex of Household Head**				
Male^Ref.^	1		1	
Female	1.00	[0.90,1.11]	0.83***	[0.74,0.93]
**Residing with Father**				
Living with him^Ref.^	1		1	
Staying elsewhere	1.17**	[1.05,1.30]	1.22***	[1.09,1.37]

*p < .05, **p < .01, ***p < .001, Reference = Ref.

**Figure 2.**
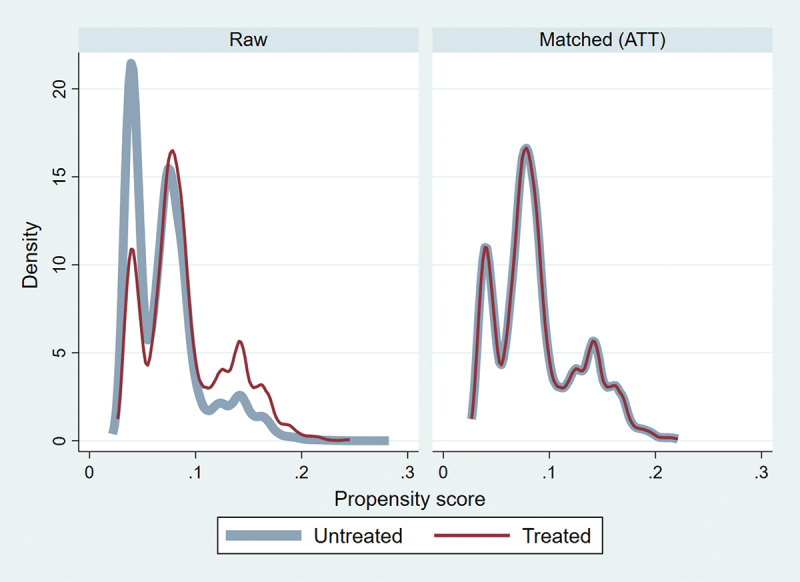
Propensity score matching using kernel matching.

**Figure 3.**
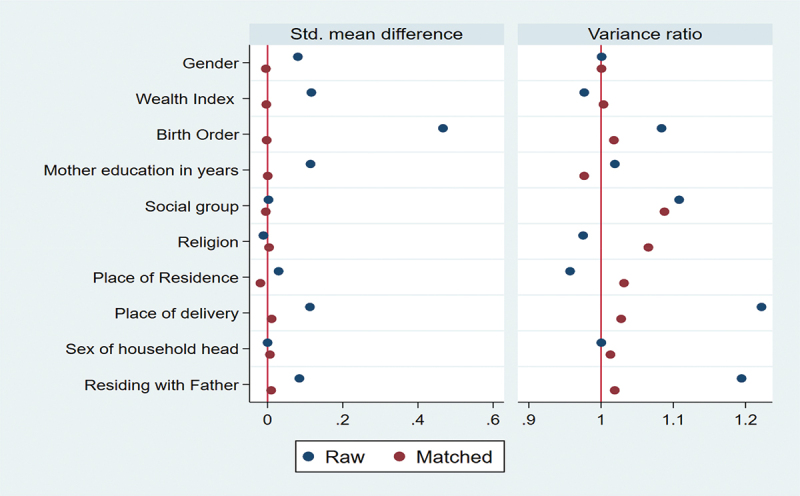
Standardized mean difference and variance ratio of covariates: raw and matched.

The results of causal estimation (Table 4) reveal that unintended pregnancy is a significant factor for zero-dose DPT vaccine among children. To know the difference in the mean outcomes of zero-dose DPT between intended pregnancy and unintended pregnancy, we used two estimates: the average treatment effect (ATE) and the average treatment effect on the treated (ATT). The estimate of average treatment effect on the treated (ATT) shows that the effect of the unintended pregnancy on the proportion of children who did not receive the first dose of the DPT vaccine was 1.3% (ATT = 0.013, 95% CI = 0.002–0.024) among children aged 12–23 months. Similarly, an estimate of the average treatment effect (ATE) shows that the effect of the unintended pregnancy on the proportion of children who did not receive the first dose of the DPT vaccine was 1.2% (ATE = 0.012, 95% CI = 0.001–0.024) among children aged 12–23 months. Figure 2 shows the balance of the propensity score distribution for all covariates between the treated and untreated groups after matching the samples. To evaluate the balance of the propensity scores in both the raw and matched data, we used kernel density plots. The results indicated that the propensity scores were imbalanced in the raw data for covariates. However, the kernel density plots for the matched data showed no significant variation in propensity scores across treatment levels, leading us to conclude that the distribution of propensity scores for covariates was well balanced after matching. Figure 3 reports the means and variances of the covariates for the treated and the untreated before and after matching.

**Table 4.** Treatment effect of unwanted birth on zero-dose of DPT vaccine in India, 2021.

Zero-dose of DPT		Coef.	Std. Err.	t	P>t	[95% Conf.	Interval]
Propensity-score kernel matching							
Unwanted pregnancy	**ATE**	**0.012**	**0.006**	**2.080**	**0.037**	**0.001**	**0.024**
Yes	Y1(ATE)	0.083	0.006	14.520	0.000	0.072	0.094
No	Y0(ATE)	0.071	0.001	54.380	0.000	0.068	0.074
Propensity-score kernel matching							
Unwanted pregnancy	**ATT**	**0.013**	**0.006**	**2.380**	**0.017**	**0.002**	**0.024**
Yes	Y1(ATT)	0.092	0.005	17.810	0.000	0.082	0.102
No	Y0(ATT)	0.078	0.002	36.620	0.000	0.074	0.083

## Discussion

Zero-dose has emerged as a key metric of childhood immunization performance, gaining prominence in the national agendas, IA 2030 framework, and Gavi’s 5.0 strategy.^39^ This study aimed to look beyond the determinants of zero-dose and explore the association between children from unintended pregnancy and zero-dose DPT vaccine among children aged 12–23 months in India. The study findings underscore the significant association between unintended pregnancy and zero-dose of the DPT vaccine. The findings highlight the higher prevalence of zero-dose vaccination among children born to mothers from unintended pregnancies. This disparity among caregivers in vaccinating their children signals the importance and necessity of considering ‘unintended pregnancy’ as a key factor contributing to the zero-dose cohort. This is the first of its kind study to explore the association between unintended children and zero-dose DPT vaccine, demonstrating its novelty in the domain of immunization research. The possible exploration and explanation of the association between a child born from unintended pregnancy and not receiving the first dose of the DPT vaccine have not been studied so far. The results provide sufficient evidence that unintended children could be a potential risk factor for zero-dose DPT vaccine in LMICs.

The prevalence of zero-dose differs considerably among children having birth from unintended pregnancy across 36 states/UTs in India. The prevalence of zero-dose DPT vaccine shows that the percentage of zero-dose children for the DPT vaccine is lowest in Puducherry (0.9%) and highest in Meghalaya (18.2%) state of India. The range from ~1 to 18% is significant, which calls for tailored interventions in areas with higher zero-dose prevalence. The following five highest zero-dose states are – Nagaland (17%), Mizoram (14.7%), Arunachal Pradesh (13.5%), Assam (10.3%), and Uttar Pradesh (10.0%). The national prevalence of zero-dose stands at 6.8%, and 13 states/UTs have zero-dose DPT that is either equal to or higher than the national average. Interestingly, the NFHS-5 data show that the states having high Total Fertility Rates (TFR) more than the replacement level of fertility have high zero-dose children. High TFR indicates mothers’ inadequate knowledge of maternal, child, and family planning services, inadequate access, and insufficient utilization.^40^

The finding of this research highlights several individual and household-level factors affecting zero-dose prevalence among children from unintended pregnancy. The wealth index was significantly associated with zero-dose of the DPT vaccine. The children from poor households had a higher odds ratio for the zero-dose of DPT as compared with children from richer households. These findings align with previous research indicating an association between higher birth order and missed vaccination among children.^26,27,37^ Likewise, our finding shows that a mother’s education level is a significant predictor of zero-dose children. Findings from previous studies have also demonstrated an association between uneducated mothers with a higher probability of having zero-dose children and partially immunized children.^17,26,33^ Place of residence was strongly associated with zero-dose DPT vaccine among children aged 12–23 months – children living in urban areas had higher chances of being zero-dose DPT vaccine than urban counterparts. These findings are not consistent with previous studies from India, wherein zero-dose children were more prevalent in rural areas (NFHS 1–4); however, the pace of progress in immunization coverage has been lesser in urban than rural regions.^26^

Similarly, we found a very interesting result at the household level – households with a female head had lower odds of zero-dose children. These findings are consistent with past studies where the odds of having zero-dose children were lower in households with a female head.^17^ This could be attributed to the fact that female-headed households have strong interpersonal communication with frontline health workers since most of the front-line workers are females, and they can efficiently communicate regarding the health and well-being of the household members and the immunization of their children. Women often play a central role in child-rearing and may have better access to health information or be proactive in seeking healthcare services for their children. Additionally, female heads of households may be more empowered to make healthcare decisions independently, reducing gender-related barriers to immunization. These, however, could differ across communities in the country and be influenced by myriad social norms and practices. Our analysis indicated that place of delivery is a strong predictor of the zero-dose DPT vaccine and the findings are aligned with the previous studies – children who were delivered at home had a higher probability of the zero-dose DPT.^26,41^ A study from India and Nepal based on Demographic and Health Survey (DHS) data found similar results suggesting unintended children who did not receive all recommended basic vaccinations along with inadequate prenatal care, were more likely to have a history of home or non-institutional births and also suffered from stunting.^37,42,43^ The findings of both univariate and multivariate logistic regression significantly demonstrated that unintended children were associated with a higher odds ratio for zero-dose of DPT compared with children born from planned pregnancy. Previous research also suggests higher odds of receiving partial or no vaccination for children who were unwanted than those who were planned.^42^

NFHS suggests significant improvement in institutional births and antenatal care over the years in India. However, the result of the PSM shows the treatment effect ‘assuming unwanted births are untreated,’ the unwanted children were statistically 1.3% times more likely to be unvaccinated for the first dose of DPT compared to planned children. There can be several potential reasons; however, the prime reason could be parents/caregivers being less vigilant about preventive healthcare services following immunization for children from unintended pregnancy compared to their children born from planned or intended pregnancy. Literature also suggests that the proportion of unintended pregnancies is higher in mothers who are not well equipped with information and lack autonomy and decision-making abilities.^44^ Women’s autonomy in reproductive decision-making and access to healthcare services play a key role in determining child vaccination and its completeness. Improving access to high-quality contraceptive services and fulfilling unmet needs for contraception can help reduce unintended pregnancies and improve child vaccination rates. Many times, these mothers and couples are in their early adulthood and sometimes late adolescent years – hence deprived of the necessary knowledge regarding immunization and preventive health services.^45^ An unintended birth largely highlights negligence toward health-seeking behaviors, and the impact is not limited to full immunization – it also affects the exclusive breastfeeding of children, nutritional status, and cognitive development. The mothers are less likely to practice exclusive breastfeeding, and the children are more likely to be underweight and stunted.^33^

In addition, our analysis reveals that 7.74% of children aged 12–23 months were born from unintended pregnancy in India. The percentage of total zero-dose (6.8%) is very close to the percentage of children born from unintended pregnancy (7.7%). A longitudinal study from India demonstrated that out of an approximately 4000 cohort of children aged 0–5 years, about 62% were unintended, and among them 76% were not fully immunized. A cohort study from India also established that unintended children were less likely to receive all routine vaccines.^33^ These insights point out a fundamental approach in public health – the ‘continuum of care’ (CoC) – that ensures mothers-to-be and newborn children receive quality maternal and newborn health services in time.^46^ This includes access to antenatal care during pregnancy, institutional deliveries by skilled birth attendants, childhood immunization, postnatal care including family planning services, and emergency obstetric and newborn care. These services are provided in a seamless continuum spanning home-based care, community, and health centers and need to be maintained throughout the reproductive lifecycle of a woman – adolescence, pregnancy, childbirth, postnatal, and childhood. Utilization of each level of the continuum determines the utilization of the next service and so forth, including immunization.^47^ A previous study in India found a significant association between mothers’ CoC and their children’s immunization status as the presence of CoC ensured their children were fully immunized.^48^ Another study illustrated that unintended children were associated with inadequate rotavirus immunization and a lower probability of attending four or more antenatal care – emphasizing the need for CoC.^36,49^

Studies have also highlighted that mother with unwanted births has poor quality relationships with their children, with a likelihood to continue even later in adulthood.^31,50^ Unwanted births have a risk for poor maternal mental health, depression, parenting stress, psychological well-being, and life satisfaction. ^51–53^ Hence, all these consequences collectively alter the mother’s attitude and behavioral relation with childcare and the utilization of preventive health services. The decision-making around child healthcare, including vaccination, is influenced if the pregnancy is planned or not. When a pregnancy is planned, parents are more likely to engage in proactive healthcare-seeking behaviors, including timely initiation of vaccinations for their children. Unintended pregnancies may result in less attention to the child’s health and lower utilization of healthcare services, including vaccination. Mothers with unintended pregnancies may not have planned for a child and may not have the necessary knowledge about the importance of vaccinations and the schedule for administering them. Parents with unintended pregnancies may not have the same level of motivation to ensure that their child receives all the recommended vaccinations. They may feel overwhelmed or unprepared for parenthood, resulting in a lack of proactive healthcare-seeking behavior. This can lead to left-out or incomplete vaccination schedules for their child.

Developed nations have effectively implemented comprehensive maternal and child health programs that integrate antenatal care, family planning, and immunization services within a single framework. This holistic approach ensures ongoing engagement with healthcare systems and significantly reduces the risk of children missing vaccinations. To address similar challenges, India is also on the way to strengthening its primary healthcare infrastructure, expanding access to family planning services, and integrating maternal and child health programs through broader health system reforms focusing on the first 1000 days. Tailored interventions, such as combining immunization visits with family planning counseling, can help close service gaps. Utilizing postnatal visits to deliver both immunization and family planning services can also reduce missed opportunities, leading to higher vaccination coverage and better maternal health outcomes with a more holistic approach to maternal and child health – making the Continuum of Care approach a priority and feasible. This will reduce the burden on caregivers and ensure compliance. The results of this paper underscore the need to consider unintended pregnancy as a significant factor in zero-dose prevalence, not only in India but also in other LMICs with similar challenges and health systems. By translating these insights into other regions with high zero-dose prevalence, the countries can leverage this understanding and concepts to develop more targeted and integrated interventions for improving childhood immunization rates and reducing healthcare disparities.

## Limitation

This study provides crucial evidence demonstrating the link between unintended births and zero-dose immunization. However, the study has a few limitations that need to be addressed. Primarily, the study’s reliance on cross-sectional household survey data might not present an accurate picture and could be susceptible to recall bias. Utilizing longitudinal data could offer more conclusive evidence of this association, given the extended period from family planning and pregnancy intendedness to a child’s first year of life, during which decisions may be influenced by changing behaviors. Additionally, the journey to full immunization requires multiple visits to health centers, and longitudinal data may capture the behavioral change and pattern, more precisely, in the long run. Given that unmet needs for contraception could influence unintended pregnancies and subsequent health-seeking behaviors, this aspect can be considered a limitation of the study. A comprehensive, in-depth qualitative study is needed to thoroughly understand the relationship between unintended births and zero-dose DPT immunization. Future research should also prioritize longitudinal studies and detailed qualitative analyses to provide a clearer and more comprehensive understanding of unintended pregnancy and the unmet need for contraception and strategies to overcome the community.

## Conclusion

In conclusion, the present study provides very clear evidence that childbirth from unintended pregnancy has an adverse impact on the uptake of child immunization services. The findings establish a link between unintended birth and the zero-dose of the DPT vaccine in India. The findings suggest that curbing the burden of unintended pregnancy could reduce the burden of zero-dose children. Efforts should be made to improve access to family planning services for planned pregnancy, increase awareness about the importance of preventive healthcare services like vaccinations, and provide support to families with unintended pregnancies to ensure that all children receive the recommended vaccinations.

## Data Availability

The data are available in the public domain and can be downloaded upon request. https://dhsprogram.com/data/available-datasets.cfm.

## Biographical note

I am a researcher with over a decade of experience in public health and social science research. He holds an M.Phil. in Population Studies and a Master’s in Population Studies from the International Institute for Population Sciences (IIPS), Mumbai. I have extensive expertise in data analysis, monitoring and evaluation, and program management, focusing on public health indicators and immunization.

The professional journey includes key roles such as Program Manager at Immunization Technical Support Unit, where I led data research and publications on child immunization and COVID-19 vaccination coverage. My research has made significant contributions to the field of public health, evidenced by his numerous publications in esteemed journals. My notable works include studies on low birth weight, the India Patriarchy Index, vaccine hesitancy, and the Impact of vaccines.

My academic and professional experiences are complemented by my participation in national and international conferences, where I presented my findings on gender policy, public health, and migration issues. My dedication to advancing public health research and policy has positioned him as a vital contributor to India’s immunization landscape.

## References

[1] Machado AA, Edwards SA, Mueller M, Saini V. Effective interventions to increase routine childhood immunization coverage in low socioeconomic status communities in developed countries: a systematic review and critical appraisal of peer-reviewed literature. Vaccine. 2021;39(22):2938–11. doi:10.1016/j.vaccine.2021.03.088. 33933317

[2] Biset G, Woday A, Mihret S, Tsihay M. Full immunization coverage and associated factors among children age 12-23 months in Ethiopia: systematic review and meta-analysis of observational studies. Hum Vaccines & Immunotherapeutics. 2021;17(7):2326–2335. doi:10.1080/21645515.2020.1870392. PMC8189140 33760689

[3] Pichichero ME. Challenges in vaccination of neonates, infants and young children. Vaccine. 2014;32(31):3886–3894. doi:10.1016/j.vaccine.2014.05.008. 24837502 PMC4135535

[4] Sáfadi MAP. The importance of immunization as a public health instrument. Jornal de Pediatria. 2023;99:91–3. SciELO Brasil. doi:10.1016/j.jped.2022.12.003. PMC10066437 36581310

[5] UNICEF. Child mortality report. 2023 [accessed 2024 June 5]. https://childmortality.org/wp-content/uploads/2024/03/UNIGME-2023-Child-Mortality-Report.pdf.

[6] Black RE, Levin C, Walker N, Chou D, Liu L, Temmerman M. Reproductive, maternal, newborn, and child health: key messages from disease control priorities 3rd edition. The Lancet. 2016;388(10061):2811–2824. doi:10.1016/S0140-6736(16)00738-8. 27072119

[7] Lindstrand A, Cherian T, Chang-Blanc D, Feikin D, O’Brien KL. The world of immunization: achievements, challenges, and strategic vision for the next decade. The J Infect Dis. 2021;224(Supplement_4):452–467. doi:10.1093/infdis/jiab284. PMC8482029 34590130

[8] WHO. Vaccines and immunization. 2024 [accessed 2024 June 15]. https://www.who.int/health-topics/vaccines-and-immunization#tab=tab_1.

[9] WHO. Immunization coverage. 2022 [accessed 2024 June 15]. https://www.who.int/news-room/fact-sheets/detail/immunization-coverage.

[10] WHO. Immunization agenda 2030: a global strategy to leave no one behind. 2020. https://cdn.who.int/media/docs/default-source/immunization/strategy/ia2030/ia2030-draft-4-wha_b8850379-1fce-4847-bfd1-5d2c9d9e32f8.pdf. 10.1016/j.vaccine.2022.11.042 39004466

[11] Wonodi C, Farrenkopf BA. Defining the zero dose child: a comparative analysis of two approaches and their impact on assessing the zero dose burden and vulnerability profiles across 82 low-and middle-income countries. Vaccines. 2023;11(10):1543. doi:10.3390/vaccines11101543. 37896946 PMC10611163

[12] Muhoza P. Routine vaccination coverage—worldwide, 2020. MMWR Morbidity And Mortal Wkly Report. 2021;70. 10.15585/mmwr.mm7043a1 PMC8553029 34710074

[13] Ram U, Ram F. Demographic transition in India: insights into population growth, composition, and its major drivers. Oxford Research Encyclopedia of Global Public Health. 2021 Apr 26 [accessed 2024 Oct 21]. https://oxfordre.com/publichealth/view/10.1093/acrefore/9780190632366.001.0001/acrefore-9780190632366-e-223.

[14] Kumar P, Chakraborty AB, Dhandore S, Dhalaria P, Singh AK, Agarwal D, Taneja G, Priyadarshini P, Jain P, Bahl V. Balancing routine and pandemic: the synergy of India’s universal immunization program and COVID-19 vaccination program. Vaccines. 2023;11(12):1776. doi:10.3390/vaccines11121776. 38140180 PMC10747509

[15] Anderson RM, May RM. Vaccination and herd immunity to infectious diseases. Nature. 1985;318(6044):323–329. doi:10.1038/318323a0. 3906406

[16] Hogan D, Gupta A. Why reaching zero-dose children holds the key to achieving the sustainable development goals. Vaccines. 2023;11(4):781. doi:10.3390/vaccines11040781. 37112693 PMC10142906

[17] Biks GA, Shiferie F, Tsegaye DA, Asefa W, Alemayehu L, Wondie T, Zelalem M, Lakew Y, Belete K, Gebremedhin S. High prevalence of zero-dose children in underserved and special setting populations in Ethiopia using a generalize estimating equation and concentration index analysis. BMC Public Health. 2024;24(1):592. doi:10.1186/s12889-024-18077-w. 38395877 PMC10893596

[18] World Health Organization. Immunization Agenda 2030. 2020 [accessed 2024 July 15]. https://www.who.int/docs/default-source/immunization/strategy/ia2030/ia2030-document-en.pdf.

[19] O’Brien KL, Lemango E, Nandy R. The immunization agenda 2030: a vision of global impact, reaching all, grounded in the realities of a changing world. Ssrn J. 2022; doi:10.2139/ssrn.3830709. PMC9754085 36528445

[20] Sokhey J, Kim-Farley RJ, Bhargava I. The expanded programme on immunization: a decade of progress in India. Ann Trop Paediatr. 1989;9(1):24–29. doi:10.1080/02724936.1989.11748590. 2471439

[21] United Nations Children’s Fund. The State of the World’s Children 2023: For every child, vaccination. Florence: UNICEF Innocenti – Global Office of Research and Foresight; 2023.

[22] Iips ICF. National family health survey (NFHS-5): 2019-21 India. Mumbai: International Institute for Population Sciences (IIPS); 2021.

[23] Bergen N, Cata-Preta BO, Schlotheuber A, Santos TM, Danovaro-Holliday MC, Mengistu T, Sodha SV, Hogan DR, Barros AJD, Hosseinpoor AR. Economic-related inequalities in zero-dose children: a study of non-receipt of diphtheria–tetanus–pertussis immunization using household health survey data from 89 low-and middle-income countries. Vaccines. 2022;10(4):633. doi:10.3390/vaccines10040633. 35455382 PMC9028918

[24] Cata-Preta BO, Santos TM, Wendt A, Hogan DR, Mengistu T, Barros AJD, Victora CG. Ethnic disparities in immunisation: analyses of zero-dose prevalence in 64 countries. BMJ Global Health. 2022;7(5):e008833. doi:10.1136/bmjgh-2022-008833. PMC9114867 35577393

[25] Rajpal S, Kumar A, Johri M, Kim R, Subramanian SV. Patterns in the prevalence of unvaccinated children across 36 states and Union territories in India, 1993-2021. JAMA Network Open. 2023;6(2):2254919–2254919. doi:10.1001/jamanetworkopen.2022.54919. PMC9918883 36763362

[26] Johri M, Rajpal S, Subramanian SV. Progress in reaching unvaccinated (zero-dose) children in India, 1992–2016: a multilevel, geospatial analysis of repeated cross-sectional surveys. The Lancet Global Health. 2021;9(12):1697–1706. doi:10.1016/S2214-109X(21)00349-1. 34798029

[27] Taneja G, Datta E, Sapru M, Johri M, Singh K, Jandu HS, Das S, Ray A, Laserson K, Dhawan V. An equity analysis of zero-dose children in india using the national family health survey data: status, challenges, and next steps. Cureus. 2023;15(2). doi:10.7759/cureus.35404. PMC9963392 36851944

[28] Santos TM, Cata-Preta BO, Wendt A, Arroyave L, Hogan DR, Mengistu T, Barros AJD, Victora CG. Religious affiliation as a driver of immunization coverage: analyses of zero-dose vaccine prevalence in 66 low- and middle-income countries. Front Publ Health. 2022;10:977512. doi:10.3389/fpubh.2022.977512. PMC9642099 36388274

[29] Santos TM, Cata-Preta BO, Mengistu T, Victora CG, Hogan DR, Barros AJD. Assessing the overlap between immunisation and other essential health interventions in 92 low-and middle-income countries using household surveys: opportunities for expanding immunisation and primary health care. EClinicalMedicine. 2021;42:101196. doi:10.1016/j.eclinm.2021.101196. 34805814 PMC8585628

[30] Wendt A, Santos TM, Cata-Preta BO, Arroyave L, Hogan DR, Mengistu T, Barros AJD, Victora CG. Exposure of zero-dose children to multiple deprivation: analyses of data from 80 low-and middle-income countries. Vaccines. 2022;10(9):1568. doi:10.3390/vaccines10091568. 36146646 PMC9502633

[31] Klima CS. Unintended pregnancy: consequences and solutions for a worldwide problem. J Nurse-Midwifery. 1998;43(6):483–491. doi:10.1016/S0091-2182(98)00063-9. 9871381

[32] Nelson HD, Darney BG, Ahrens K, Burgess A, Jungbauer RM, Cantor A, Atchison C, Eden KB, Goueth R, Fu R. Associations of unintended pregnancy with maternal and infant health outcomes: a systematic review and meta-analysis. Jama. 2022;328(17):1714–1729. doi:10.1001/jama.2022.19097. 36318133 PMC9627416

[33] Chowdhury P, Garg MK, Sk MIK. Does mothers’ pregnancy intention affect their children’s preventive and curative care in India? Evidence from a longitudinal survey. BMJ Open. 2021;11(4):e042615. doi:10.1136/bmjopen-2020-042615.

[34] Doskoch P. Unplanned pregnancy linked to poor child health in India. Int Perspectives On Sexual And Reprod Health. 2012;38(4):223–224.

[35] Gipson JD, Koenig MA, Hindin MJ. The effects of unintended pregnancy on infant, child, and parental health: a review of the literature. Stud in Fam Plann. 2008;39(1):18–38. doi:10.1111/j.1728-4465.2008.00148.x. 18540521

[36] Hajizadeh M, Nghiem S. Does unwanted pregnancy lead to adverse health and healthcare utilization for mother and child? Evidence from low- and middle-income countries. Int J Public Health. 2020;65(4):457–468. doi:10.1007/s00038-020-01358-7. 32270238 PMC7275006

[37] Singh A, Singh A, Mahapatra B. The consequences of unintended pregnancy for maternal and child health in rural India: evidence from prospective data. Matern Child Health J. 2013;17(3):493–500. doi:10.1007/s10995-012-1023-x. 22527770

[38] Jann B. KMATCH: Stata module for multivariate-distance and propensity-score matching. 2017. https://econpapers.repec.org/software/bocbocode/s458346.htm.

[39] Patel C, Rendell N, Sargent GM, Ali A, Morgan C, Fields R, Sheel M. Measuring national immunization system performance: a systematic assessment of available resources. Glob Health Sci Pract. 2023;11(3):e220055. doi:10.9745/GHSP-D-22-00555. 37348935 PMC10285727

[40] Singh S, Shekhar C, Bankole A, Acharya R, Audam S, Akinade T. Key drivers of fertility levels and differentials in India, at the national, state and population subgroup levels, 2015–2016: an application of Bongaarts’ proximate determinants model. PloS One. 2022;17(2):e0263532. 35130319 10.1371/journal.pone.0263532 PMC8820640

[41] Farrenkopf BA, Zhou X, Shet A, Olayinka F, Carr K, Patenaude B, Chido-Amajuoyi OG, Wonodi C, Harapan H. Understanding household-level risk factors for zero dose immunization in 82 low- and middle-income countries. PLOS ONE. 2023;18(12):e0287459. doi:10.1371/journal.pone.0287459. 38060516 PMC10703331

[42] Singh A, Chalasani S, Koenig MA, Mahapatra B. The consequences of unintended births for maternal and child health in India. Popul Stud. 2012;66(3):223–239. doi:10.1080/00324728.2012.697568. 22783949

[43] Singh A, Singh A, Thapa S. Adverse consequences of unintended pregnancy for maternal and child health in Nepal. Asia Pac J Public Health. 2015;27(2):1481–1491. doi:10.1177/1010539513498769. 24097931

[44] Dalmijn EW, Visse MA, van Nistelrooij I. Decision-making in case of an unintended pregnancy: an overview of what is known about this complex process. J Psychosomatic Obstet & Gynecol. 2024;45(1):2321461. doi:10.1080/0167482X.2024.2321461. 38469857

[45] Balgovind P, Mohammadnezhad M. Factors affecting childhood immunization: thematic analysis of parents and healthcare workers’ perceptions. Hum Vaccines & Immunotherapeut. 2022;18(6):2137338. doi:10.1080/21645515.2022.2137338. PMC9746479 36494999

[46] UNICEF. 2020 [accessed 2024 Jul 2]. https://www.unicef.org/india/what-we-do/maternal-health.

[47] Phway P, Kyaw AT, Mon AS, Mya KS. Continuum of care of mothers and immunization status of their children: a secondary analysis of 2015-2016 myanmar demographic and health survey. Public Health In Pract (Oxford, Engl). 2022;4:100335. doi:10.1016/j.puhip.2022.100335. PMC9640345 36389260

[48] Usman M, Anand E, Siddiqui L, Unisa S. Continuum of maternal health care services and its impact on child immunization in India: an application of the propensity score matching approach. J Biosocial Sci. 2021;53(5):643–662. doi:10.1017/S0021932020000450. 32830633

[49] Echaiz J, Blas M, Kancherla V. Unintended pregnancy and its impact on childhood rotavirus immunization in Peru. Rev Panam Salud Publica. 2018;42:e96. doi:10.26633/RPSP.2018.96. 31093124 PMC6386012

[50] Barber JS, Axinn WG, Thornton A. Unwanted childbearing, health, and mother-child relationships. J Health And Soc Behav. 1999;40(3):231–257. doi:10.2307/2676350. 10513146

[51] Bahk J, Yun SC, Kim Y, Khang YH. Impact of unintended. Pregnancy on maternal mental health: a causal analysis using follow up data of the panel study on Korean children (PSKC). BMC Pregnanc Childbirth. 2015;15(1):1–12. doi:10.1186/s12884-015-0505-4. PMC4387588 25881099

[52] Hardee K, Eggleston E, Wong EL, Hull TH Unintended pregnancy and women’s psychological well-being in Indonesia. J Biosoc Sci. 2004;36(5):617–626. doi:10.1017/S0021932003006321. 15446355

[53] Orr ST, Miller CA. Unintended pregnancy and the psychosocial well-being of pregnant women. Women’s Health Issues. 1997;7(1):38–46. doi:10.1016/S1049-3867(96)00017-5. 9009862

